# Interleukin-6 and the determinants of severe COVID-19: A retrospective cohort study

**DOI:** 10.1097/MD.0000000000036037

**Published:** 2023-11-10

**Authors:** Wael Hafez, Prashant Nasa, Ahmed Khairy, Mohan Jose, Mahmoud Abdelshakour, Sabah Ahmed, Fatema Abdulaal, Nivedita Nair, Mohammad Ahmad, Vanya Jalal Rashid, Youmna Ayman, Steffi John, Osman Fdl Alla, Reham Abu Shady, Ahmed Ali Mohamed, Rami Soliman, Simon Nader

**Affiliations:** a Internal Medicine, NMC Royal Hospital, Khalifa City, Abu Dhabi, United Arab Emirates; b Internal Medicine Department, The Medical Research Division, The National Research Center, Cairo, Egypt; c NMC Specialty Hospital, Al Nahda, Dubai, United Arab Emirates; d Intensive Care Unit, Theodor Bilharz Research Institute, El Warraq, Giza Governorate, Egypt; e National Institute of chest and Allergy, Cairo, Egypt.

**Keywords:** acute respiratory distress syndrome, COVID-19, cytokine storm, interleukin-6, obesity, severity

## Abstract

Cytokines, notably interleukin-6 (IL-6), increase considerably in patients with severe corona virus disease 2019 (COVID-19). This vigorous immune response may cause end-organ failure or death; hence, measuring IL-6 in the context of patient characteristics may help predict outcomes and encourage early comprehensive therapy. This study investigated the association between serum IL-6 levels, COVID-19 severity, and demographic, clinical, and biochemical characteristics. COVID-19 inpatients in NMC hospitals were investigated between November 2020 and November 2021. Several patient variables related to serum IL-6 and COVID-19 severity have been examined. The study included 374 COVID-19 inpatients, 235 of whom had severe disease with a median age of 51. The elderly had an increased risk of severe COVID-19 (73.8%) compared with young adults (71%), with higher white blood cells, D-dimer, Lactate dehydrogenase, creatinine, ferritin, prothrombin time, Procalcitonin, and fibrinogen levels (*P* < .001). C-reactive protein, troponin, intensive care unit admission, disease severity score, and mortality were significantly associated with higher serum IL-6 levels (*P* = .05) in the univariate analysis, but this significance disappeared in the multivariate analysis. IL-6, along with other demographic and clinical variables affected COVID-19 severity. These characteristics may predict patients at risk of severe disease and assist in establishing early comprehensive disease outcome strategies. Large-scale clinical research is needed to emphasize IL-6 and COVID-19.

## 1. Introduction

On December 31, 2019, the World Health Organization (WHO) China Country Office was informed of cases of pneumonia of unknown etiology detected in Wuhan City, Hubei Province, China, which was later defined as coronavirus disease 2019 (COVID-19).^[1]^ In the early stages of COVID-19, patients often experience flu-like symptoms, such as fever, cough, fatigue, body aches, sore throat, and headache. However, it is crucial to note that COVID-19 can present with a wide range of symptoms and some individuals may remain asymptomatic. This variability underscores the need for a comprehensive understanding of COVID-19’s diverse manifestations.^[2,3]^ On the other hand, others may develop more severe symptoms, such as pneumonia, acute respiratory distress syndrome (ARDS), or multiorgan dysfunction. The wide spectrum of COVID-19 severity makes it a complex and unpredictable disease and the course of the illness can vary significantly among individuals. Many patients with COVID-19 develop ARDS, which is occasionally accompanied by multiorgan failure.^[4]^ Comorbidities such as diabetes mellitus, cardiovascular disease, and hypertension increase the risk of mortality due to COVID-19.^[5]^ A cytokine storm, defined by the overproduction of molecules associated with inflammation, such as interleukin-6 (IL-6), C-reactive protein (CRP), and ferritin, has been proposed as the pathophysiology of the severe form of COVID-19.^[6]^ One of the most crucial components of the cytokine network engaged in the immune defense system is that the IL-6.^[7]^ The IL-6 pathway is associated with immune control and immunological dysregulation. Increased IL-6 levels also reflect the stress response observed during viral infection or acute inflammation.^[8]^ Recent research has shown that IL-6 controls several metabolic processes including glucose intake, glycolysis, fatty acid oxidation, and oxidative phosphorylation. Additionally, numerous diseases caused by viral infections have significantly higher levels of IL-6 expression, which is positively correlated with disease severity.^[9]^ Inflammatory cytokine levels have been shown to be considerably higher in severe COVID-19 cases than in mild cases, with IL-6 being a critical mediator of hyperinflammation.^[6]^ The goal of this study was to examine the potential factors that influence serum interleukin-6 levels, as well as their relationship to COVID-19 disease outcomes in an ethnically diverse population living in the United Arab Emirates.

## 2. Methods

This was an observational retrospective study. This study was conducted on patients previously admitted to 2 major tertiary care hospitals in the United Arab Emirates between November 2020 and November 2021. The inclusion criteria of our study consisted of COVID-19 patients aged > 18 years who were collected from electronic medical records and included demographic, clinical assessment, laboratory measurements, imaging, and COVID-19-related outcomes. Complete blood count, liver function test results, and C-reactive protein (mg/L), D-dimer (ng/mL), fibrinogen (mg/dL), ferritin (ng/mL), and IL-6 (pg/mL) levels were also collected. A real-time reverse transcription-polymerase chain reaction test on a nasopharyngeal swab sample was used to detect severe acute respiratory syndrome coronavirus 2. An Xybio Extraction Kit (Korea) was used for RNA extraction. For reverse transcription-polymerase chain reaction analysis, Bio-Rad Cycler PCR (USA) was used in conjunction with Solgent 2019-nCoV Real-Time Reverse Transcription PCR Kit. A CFX-96 plate reader (Bio-Rad) was used for viral detection. Cycle threshold values (Ct values) > 40 were considered positive, whereas computed tomography values < 40 were considered negative. Data for chest radiography and/or chest computed tomography were obtained from patients at the time of admission and throughout the follow-up period. Severe COVID-19 outcomes were assessed using the WHO Severity Scale for COVID-19, and clinical progress was assessed using the WHO recommendations. The IL-6 test was performed by measuring serum IL-6 concentrations using an enzyme-linked immunosorbent assay. The tests were conducted at the UAE National Reference Laboratory. LabCorp Burlington, 1447 York Cort, Burlington, NC 27215-3361, created the test. In this study, the normal serum IL-6 levels ranged from 0.0 to 15.5 pg/mL. Only patients who met the inclusion criteria were included in this study. In total, 374 patients were recruited for this study. Data were analyzed using SPSS version 28 (Statistical Package for the Social Sciences). *P* values equal to or < .05 were deemed significant. Continuous data are presented as means and standard deviations for normally distributed data and medians and interquartile ranges for non-normally distributed data, while categorical data are presented as frequencies and percentages. The Kolmogorov-Smirnov test was employed to assess the real distribution of continuous data. In addition, we used binary logistic regression to identify predictors that were genuinely related to a higher level of IL-6 (above 48.8 pg/mL) (dependent variable) using the following 2 models (1st curve odds ratio (no-adjusting), 2nd correcting for all covariates).

## 3. Results

### 3.1. Severity index based on base line characteristic

This retrospective analysis comprised 374 COVID-19 patients. Age, IL-6 level (pg/mL), comorbidities including hypertension and diabetes mellitus, intensive care unit (ICU) admission, high-flow, noninvasive high-flow oxygen, noninvasive ventilation, and invasive mechanical ventilation all had a statistically significant relationship with the severity index (*P* = .01). The median age of the patients with a severity index was 51 years, with the elderly accounting for 73.8% of severe cases, whereas the majority of non severe cases (71%) were young. Almost all of the study population (94%) had IL-6 levels over 43.5, indicating severe COVID-19, whereas 29.8% had IL-6 levels below 43.5, indicating non severe COVID-19. (Table 1).

**Table 1:** Severity index based on base line characteristic.

Variable	Categories	Severity index				Missing		*P* value
		Severe		Non severe				
		N	%	N	%	N	%	
Age (median - interquartile range)		(51–22)		(39–35)		0	0%	<.001
Age group	Child	0	0.0%	29	100.0%	0	0%	<.001
	Young adults	9	29.0%	22	71.0%			
	Middle-aged adults	68	68.0%	32	32.0%			
	Old-aged adults	158	73.8%	56	26.2%			
Sex	Female	74	57.4%	55	42.6%	0	0%	.113
	male	161	65.7%	84	34.3%			
IL-6 level group	Under 43.5	40	70.2%	17	29.8%	266	70.6%	.001
	Above 43.5	51	94.4%	3	5.6%			
Blood group	A	8	66.7%	4	33.3%	299	79.3%	.165
	B	20	87.0%	3	13.0%			
	AB	3	60.0%	2	40.0%			
	O	23	60.5%	15	39.5%			
RH	Negative	4	50.0%	4	50.0%	301	79.8%	.168
	Positive	50	73.5%	18	26.5%			
Co morbidity hypertension	No	136	57.1%	102	42.9%	1	0.3%	.002
	Yes	99	73.3%	36	26.7%			
Co morbidity diabetes mellitus	No	133	58.3%	95	41.7%	1	0.3%	.019
	Yes	102	70.3%	43	29.7%			
Co morbidity -lung disease, asthma, COPD	No	219	63.1%	128	36.9%	3	0.8%	.640
	Yes	14	58.3%	10	41.7%			
ICU admission	No	51	27.9%	132	72.1%	2	0.5%	<.001
	Yes	183	96.8%	6	3.2%			
HF-NIV high-flow oxygen -non-invasive ventilation	No	171	55.2%	139	44.8%	14	3.7%	<.001
	Yes	50	100.0%	0	0.0%			
Invasive mechanical ventilation	No	147	51.4%	139	48.6%	1	0.3%	<.001
	Yes	87	100.0%	0	0.0%			

ICU = intensive care unit, IL-6 = interleukin-6.

### 3.2. The deference of median of severity index based on base line characteristic

Severe COVID-19 cases in the study group showed greater levels of IL-6 than non severe patients (median = 48.8, *P* = .001). Furthermore, non severe patients had lower levels of white blood cell and CRP than severe patients (median = 6.31 × 10^3/μL, *P* = .001) (median = 18 mg/L, *P* = .001). Furthermore, patients with severe COVID-19 had greater ferritin levels than non severe patients (median = 930.85 ng/mL, *P* = .001). The neutrophil count was higher in patients with severe disease (median = 85.24%, *P* = .001), whereas the lymphocyte count was considerably lower (median = 24.9%, *P* = .001). Furthermore, individuals with severe COVID-19 difficulties had higher fibrinogen levels than non severe cases (mean = 636.16, SD = 164,63 mg/dL, *P* value .001).(Table 2).

**Table 2:** The deference of median of severity index based on base line characteristics.

Variable	Severity index	Valid	Missing		*P* value
		Median - IQR	N	Percent	
IL-6 level	Severe	48.8–107.1	266	70.6%	<.001
	Non severe	11.05–19.1			
WBC	Severe	9.27–9.79	4	1.1%	<.001
	Non severe	6.31–4.75			
Hemoglobin	Severe	13.1–3.1	3	0.8%	.880
	Non severe	12.8–2.7			
Platelets	Severe	236–191.8	4	1.1%	.497
	Non severe	241–148.5			
CRP	Severe	122–143	5	1.3%	<.001
	Non severe	18–46			
D-dimer	Severe	507.5–1600	39	10.3%	<.001
	Non severe	0.61–1			
LDH	Severe	471–308	68	18.1%	<.001
	Non severe	251–133			
ALT at time of hospital admission	Severe	47–55	62	16.5%	<.001
	Non severe	34–29			
AST at time of admission	Severe	52.5–48.5	61	16.2%	<.001
	Non severe	34–28			
Creatinine	Severe	0.9–0.49	25	6.6%	<.001
	Non severe	0.78–0.39			
Neutrophil count	Severe	85.24–12.65	3	0.8%	<.001
	Non severe	68.15–24.07			
Lymphocyte count	Severe	15.3–14.6	5	1.3%	<.001
	Non severe	24.9–21.33			
NLR neutrophil to lymphocyte ratio	Severe	5.2–3.7	335	88.9%	<.001
	Non severe	2.8–3.11			
RDW-CV red blood cell width	Severe	13.7–2.5	3	0.8%	.064
	Non severe	13.4–2.3			
Ferritin	Severe	930.85–1301	49	13%	<.001
	Non severe	257–417			
PT	Severe	15–2.4	160	42.4%	<.001
	Non severe	14–2			
INR	Severe	1.13–0.22	160	42.4%	<.001
	Non severe	1.05–0.1			
Troponin I	Severe	0.02–0.07	128	33.9%	<.001
	Non severe	0.002–0.01			
Procalcitonin	Severe	0.18–0.57	100	26.5%	<.001
	Non severe	0.06–0.12			
Glucose	Severe	18–151.5	151	40.1%	<.001
	Non severe	5.4 - 4			
Fibrinogen	Severe	636.16–164.63	186	49.3%	<.001
	Non severe	489.99–152.06			

CRP = C-reactive protein, IL-6 = interleukin-6, INR = International normalized ratio, LDH = Lactate dehydrogenase, NLR = Neutrophil to lymphocyte ratio, PT = prothrombin time, RDW-CV = red blood cell width, WBC = white blood cell.

### 3.3. Kendall tau b correlation coefficient for all variables

There was a slight positive correlation between IL-6 levels in COVID-19 patients and CRP, fibrinogen, and procalcitonin levels (RS = 0.25, *P* value .001), (RS = 0.31, *P* value .001), and (RS = 0.24, *P* value .001). There was a statistically significant moderate correlation between CPR and D-DIMER, Lactate dehydrogenase, fibrinogen, and procalcitonin (Rs = 0.32, *P* value .001), (RS = 0.49, *P* value .001), and (RS = 0.40, *P* value .001), respectively. Lactate dehydrogenase, procalcitonin, and fibrinogen levels, however, had statistically significant weak-to-moderate correlations (RS = 0.29, *P* value .001) and (RS = 0.34, *P* value .001), respectively. (Table 3).

**Table 3:** Kendall tau b correlation coefficient for all variables.

		IL-6 level	CRP	D-dimer	LDH	Fibrinogen	Procalcitonin
IL-6 level	RS	_	0.253	0.119	0.115	0.312	0.245
	*P* value		<.001	.066	.075	.001	<.001
CRP	RS	_	_	0.326	0.327	0.497	0.405
	*P* value			<.001	<.001	<.001	<.001
D-dimer	RS	_	_	_	0.374	0.234	0.339
	*P* value				<.001	<.001	<.001
LDH	RS	_	_	_	_	0.344	0.290
	*P* value					<.001	<.001
Fibrinogen	RS	_	_	_	_	_	0.159
	*P* value						.007
Procalcitonin	RS	_	_	_	_	_	_
	*P* value						

CRP = C-reactive protein, IL-6 = interleukin-6, LDH = Lactate dehydrogenase.

### 3.4. Association between IL-6 of patients with COVID-19 and variables

When we performed a non-adjusted logistic regression analysis among patients with COVID-19, we discovered that 5 out of 13 predictor variables were significantly associated with IL-6 levels, including ICU admission, CRP, troponin, and severity index (*P* value < .05). Patients with CRP levels > 10 mg/L were more likely to have higher levels of IL-6 than those with CRP levels < 10 mg/L (COR = 11.52, *P* value = .022). Additionally, severe cases who were admitted to the ICU demonstrated 3.27 times greater existence of high levels of IL-6 levels than those who did not (*P* value = .006). Patients who died had a higher risk of having an IL-6 level than those who improved (COR = 14.32, *P* value = .012). Regarding troponin levels, the study population with troponin levels above 0.04 ng/mL has a higher likely to have higher levels of IL-6 than others with troponin levels below 0.04 ng/mL (COR = 3.73, *P* value = .021). (Table 4).

**Table 4:** Association between IL-6 of patients with COVID-19 and variables.

Variable	Categories	IL-6 level			
		*P* value	COR	95% CI for B	
				Lower	Upper
Age group	Young adults	Ref			
	Middle-aged adults	.634	0.679	0.138	3.342
	Old-aged adults	.205	2.667	0.586	12.145
SEX	Female	Ref			
	male	.286	0.608	0.244	1.517
CRP group	Under 10	Ref			
	Above 10	.022	11.522	1.421	93.453
D-dimer group	Under 500	Ref			
	Above 500	.304	1.481	0.7	3.133
LDH group	Under 333	Ref			
	Above 333	.593	1.262	0.538	2.956
Fibrinogen group	Under 400	Ref			
	Above 400	.777	1.312	0.200	8.624
Blood group	A	Ref			
	B	.083	0.119	0.011	1.323
	AB	.501	0.333	0.014	8.182
	O	.107	0.148	0.015	1.510
Died	No	Ref			
	Yes	.012	14.326	1.780	115.282
ICU admission	No	Ref			
	Yes	.006	3.279	1.412	7.615
Troponin group	Under 0.04	Ref			
	Above 0.04	.021	3.733	1.222	11.401
Severity index	Severe	Ref			
	Non severe	.003	0.138	0.038	0.505
RH	Negative	Ref			
	Positive	.503	0.533	0.085	3.351

COVID-19 = coronavirus disease, CRP = C-reactive protein, ICU = intensive care unit, IL-6 = interleukin-6, LDH = Lactate dehydrogenase.

### 3.5. Non-adjusted cox regression among patients with COVID-19 depending on levels of IL-6

We conducted a non-adjusted Cox regression analysis to detect the relative risk of death among COVID-19 patients based on levels of IL-6. No statistically significant associations were observed between variables. (*P* > .05), respectively. (Table 5).

**Table 5:** A non-adjusted cox regression among patients with COVID-19 depending on levels of IL-6.

Variable	Categories	DIED			
		*P* value	RR	95% CI for B	
				Lower	
Age group	Young adults	Ref			
	Middle-aged adults	.805	152.907	_	_
	Old-aged adults	.754	608.278	_	_
Sex	Female	Ref			
	Male	.985	0.961	0.017	54.355
Body mass index group	Healthy weight	Ref			
	Overweight	.300	13.992	0.095	2053.607
	Obese	.901	1.204	0.065	22.165
CRP group	Under 10	Ref			
	Above 10	.653	2043.948	_	_
D-dimer group	Under 500	Ref			
	Above 500	.366	0.358	0.039	3.309
LDH group	Under 333	Ref			
	Above 333	.705	2.096	0.045	96.716
Fibrinogen group	Under 400	Ref			
	Above 400	1	_	0.001	_
Blood group	A	Ref			
	B	.881	1.396	0.018	109.809
	AB	.210	0.030	0.001	7.249
	O	.852	0.737	0.030	17.962
IL-6 level group	Under 43.5	Ref			
	Above 43.5	.467	5.556	0.055	562.216
ICU admission	No	Ref			
	Yes	.639	3838.323	_	_
Severity index	Severe	Ref			
	Non severe	.963	3.239	_	_
RH	Negative	Ref			
	Positive	.366	2.859	0.293	27.906

COVID-19 = coronavirus disease, CRP = C-reactive protein, ICU = intensive care unit, IL-6 = interleukin-6, LDH = Lactate dehydrogenase.

## 4. Discussion

The inflammatory response against COVID-19 virus is accused of development COVID-19-related complications. Evidence suggests that an imbalanced proinflammatory immune response may contribute to the development of the most life-threatening stages of the disease. Accordingly, high levels of circulating cytokines and acute-phase reactants are associated with poor outcomes in COVID-19 patients.^[10]^

Our study found that the likelihood of experiencing severe COVID-19 disease is strongly related to age, with the elderly being considerably more likely to experience extreme disease than young people. A similar conclusion was reached in a study in Dubai, which found that the severity of COVID-19 was 4.7 times higher in older people than in young adults.^[11]^ In addition to age-related immune system alterations, this finding may also be explained by the fact that older patients tend to have more chronic illnesses (hypertension, coronary artery disease, and diabetes), which worsen patient outcomes.

Although our results revealed that the connection between gender and severe COVID-19 disease was not statistically significant, many other studies have indicated that males are more vulnerable to developing a critical disease than females.^[12,13]^ These studies attributed their findings to differences in sex hormones and immune-related genes found on the X chromosome; however, the effect of sex on the development of chronic COVID-19 is not fully understood and additional research is required. We also discovered a significant correlation between IL-6 serum levels and the severity of COVID-19, as patients with elevated IL-6 levels (above 43.5 pg/mL) were found to be 94.4 percent more likely to experience severe disease, and several studies from Finland and China support these findings.^[14,15]^ Moreover, a meta-analysis of 44 articles confirmed a link between COVID-19 severity and high IL-6 levels.^[16]^ The ability of cytokines (including IL-6) to cause epithelial and endothelial cell death and increase vascular permeability, which contributes to the development of ARDS and even death, is thought to be a strong association between cytokine storms and the progression of COVID-19 disease from moderate to severe and critical stages.^[16–18]^ We discovered that respiratory illnesses such as asthma and COPD had no effect on the severity of COVID-19 infection (*P* > .05); a similar conclusion was reported by another Danish study.^[19]^ However, a cohort study in England reported that all respiratory diseases were related to an elevated risk of hospitalization in patients with COVID-19 compared to those without respiratory diseases.^[20]^ Further research into the severity of COVID-19 infection in individuals with lung disorders, such as Bronchial Asthma and chronic obstructive pulmonary disease, is needed.

A substantial difference in various laboratory values was observed in severe cases, such as D-dimer, which increased significantly in severe cases (507.5 ng/mL) compared to non severe cases (0.61 ng/mL). A similar finding was reported by 12 of the 13 studies included in another meta-analysis study.^[21]^ Additionally, researchers were able to use D-dimer levels as a biomarker to predict the severity of COVID-19 infection and assess the need to provide more aggressive care.^[22]^ These findings support the hypothesis that COVID-19 could adversely affect the function of the hemostatic system, causing thrombophilia and an increased tendency to form clots.^[23]^ A study in New York confirmed that the elevation of both CRP and D-dimer concentrations had the greatest risk of adverse events,^[24]^ which is similar to our results that indicated a rather higher value of CRP in severe cases compared to modest COVID-19 infection (122 mg/L and 18 mg/L, respectively). The increasing levels of IL-6, which are considerably high in severe cases, play a critical role in inducing CRP synthesis.^[25]^ Another interesting finding was that the severity of the disease was associated with the levels of liver enzymes at the time of hospitalization (*P* < .001), as AST and ALT levels increased moderately in patients with severe cases compared to patients with non severe COVID-19 infection. Similarly, a meta-analysis confirmed that the increase in liver enzymes in COVID-19, even in the severe phase, was moderate in most cases.^[26]^ This elevation may not be solely the result of viral infection but may also be attributable to the cytokine storm and the use of multiple medications during hospitalization (antivirals, antibiotics, and analgesics), both of which can negatively impact liver function. Spearman correlation coefficient was used to study the associations between different laboratory variables. Our results showed a moderately positive correlation between CRP and Procalcitonin (RS = 0.405 mg/L); a similar moderate correlation was reported in a study in Serbia (RS = 0.423 mg/L).^[27]^ These 2 proteins are produced at high levels during the inflammatory response; thus, it is evident that both would elevate COVID-19 infection.

Although we discovered a statistically significant link between rising IL-6 levels and the mortality rate (COR = 14.32, *P* = .012), this relationship disappeared after correcting our data for other covariates (*P* = .99). These findings indicate that IL-6 level alone may not be an accurate predictor of mortality in COVID-19 patients. A meta-analysis of 17 studies found that IL-6 levels correlated with disease severity; however, they should not be used exclusively to predict mortality.^[28]^ Possible impact of other patient characteristics on COVID-19 disease outcomes. Recent studies have shown elevated levels of CRP, neutrophils, and lymphopenia in COVID-19 patients.^[29]^ These findings, along with the emphasis of our study on the significance of IL-6 and CRP as key inflammatory markers associated with COVID-19 severity, support the critical role of inflammation in the pathogenesis of severe COVID-19. Our study also established specific cutoff points for these markers, making them potential tools for identifying patients at risk of developing severe disease. The convergence of these results across studies reinforces the significance of these inflammatory markers as valuable indicators of COVID-19 severity and highlights their potential utility in guiding clinical decision-making.

## 5. Strength and limitations

The significance of our research lies in its focus on the severity of COVID -19 infection and its relationship with IL-6 through comparison with various demographic, clinical, and biochemical characteristics, which in turn would guide researchers to conduct future studies that could help predict patient outcomes based on these variables. Furthermore, it includes a wide variety of demographic clinical features and laboratory parameters of COVID-19 patients from a major tertiary care hospital in the UAE, which would supplement the literature with data from this location, in addition to the multiethnic group addressed in this study. This study had certain limitations that should be acknowledged. The sample size was modest, and some confounding variables, such as body mass index, could not be explored. Furthermore, missing data cannot be prevented in retrospective observational studies that use EHR data, which may skew or distort our results. Future studies with larger sample sizes and longitudinal data on disease development during hospitalization are required.

## 6. Conclusion

IL-6 has been demonstrated to be a key biomarker of COVID-19 severity, and various demographic and clinical characteristics have been linked to its levels and the severity of COVID-19 infection. Identifying these features may also enable the early identification of patients at risk for severe disease as well as the establishment of early comprehensive efforts to improve disease outcomes. Further large-scale clinical research is required to emphasize the link between IL-6 and COVID-19 outcomes.

## Acknowledgments

I would like to express my sincere gratitude to David Hardley, Alan Stewart, Prakash Janardan, Maki Hamad, Mouhamad Al Zouhby, and the NMC Clinical Research team, Gayathri Rahul, Rohit Dusane, Shailesh Perdakar, and Monica Jadhav for their help, guidance, and support.

## Author contributions

**Conceptualization:** Wael Hafez.

**Data curation:** Wael Hafez, Prashant Nasa, Ahmed Khairy, Mohan Jose, Sabah Ahmed, Fatema Abdulaal, Nivedita Nair, Mohammad Ahmad, Vanya Jalal Rashid, Youmna A Faheem, Steffi John, Osman Fdl All, Ahmed A Mohamed, Rami Soliman, Simon Nader.

**Formal analysis:** Wael Hafez, Ahmed Khairy, Mohan Jose, Mahmoud Abdelshakour, Sabah Ahmed, Fatema Abdulaal, Nivedita Nair, Vanya Jalal Rashid, Reham Abushady, Ahmed A Mohamed, Rami Soliman, Simon Nader.

**Funding acquisition:** Wael Hafez, Sabah Ahmed, Osman Fdl All.

**Investigation:** Wael Hafez, Prashant Nasa, Mohan Jose, Mahmoud Abdelshakour, Fatema Abdulaal, Nivedita Nair, Mohammad Ahmad, Vanya Jalal Rashid, Steffi John, Reham Abushady, Ahmed A Mohamed, Rami Soliman, Simon Nader.

**Methodology:** Wael Hafez, Prashant Nasa, Ahmed Khairy, Mahmoud Abdelshakour, Sabah Ahmed, Nivedita Nair, Mohammad Ahmad, Vanya Jalal Rashid, Youmna A Faheem, Steffi John, Reham Abushady.

**Project administration:** Wael Hafez.

**Resources:** Wael Hafez, Mohan Jose, Mahmoud Abdelshakour, Fatema Abdulaal, Nivedita Nair, Mohammad Ahmad, Vanya Jalal Rashid, Reham Abushady, Simon Nader.

**Software:** Wael Hafez, Sabah Ahmed, Osman Fdl All.

**Supervision:** Wael Hafez, Prashant Nasa, Ahmed Khairy, Mahmoud Abdelshakour, Sabah Ahmed, Fatema Abdulaal, Nivedita Nair, Mohammad Ahmad, Rami Soliman.

**Validation:** Wael Hafez, Simon Nader.

**Visualization:** Wael Hafez, Prashant Nasa, Steffi John.

**Writing – original draft:** Wael Hafez, Rami Soliman.

**Writing – review & editing:** Wael Hafez, Ahmed Khairy, Mohan Jose, Nivedita Nair, Mohammad Ahmad, Vanya Jalal Rashid, Youmna A Faheem, Steffi John, Osman Fdl All, Reham Abushady, Ahmed A Mohamed, Simon Nader.

## References

[1] Coronavirus Disease (COVID-19) Situation Reports. In: Coronavirus Disease (COVID-19) Situation Reports. https://www.who.int/emergencies/diseases/novel-coronavirus-2019/situation-reports. [accessed 19 Dec 2022]

[2] Aktas G. A comprehensive review on rational and effective treatment strategies against an invisible enemy; SARS Cov-2 infection. Exp Biomed Res. 2020;3:293–311.

[3] Wu Z McGoogan JM. Characteristics of and important lessons from the coronavirus disease 2019 (COVID-19) outbreak in China: summary of a report of 72 314 cases from the Chinese center for disease control and prevention. JAMA. 2020;323:1239–42. 32091533 10.1001/jama.2020.2648

[4] Lippi G Plebani M. Procalcitonin in patients with severe coronavirus disease 2019 (COVID-19): a meta-analysis. Clin Chim Acta. 2020;505:190–1. 32145275 10.1016/j.cca.2020.03.004 PMC7094472

[5] de Almeida-Pititto B Dualib PM Zajdenverg L. Severity and mortality of COVID 19 in patients with diabetes, hypertension and cardiovascular disease: a meta-analysis. Diabetol Metab Syndr. 2020;12:1–2. 32874207 10.1186/s13098-020-00586-4 PMC7456786

[6] Huang C Pranata R Lim MA. C-reactive protein, procalcitonin. D-dimer, and ferritin in severe coronavirus impact of COVID-19 on oral and dental health delivery and recommendations. DOI: http://dx. doi. org/10.5772/intechopen.;98522.

[7] Jordan SC Choi J Kim I. Interleukin-6, a cytokine critical to mediation of inflammation, autoimmunity and allograft rejection: therapeutic implications of IL-6 receptor blockade. Transplantation. 2017;101:32–44. 27547870 10.1097/TP.0000000000001452

[8] Pal M Febbraio MA Whitham M. From cytokine to myokine: the emerging role of interleukin-6 in metabolic regulation. Immunol Cell Biol. 2014;92:331–9. 24751614 10.1038/icb.2014.16

[9] Li YS Ren HC Cao JH. Roles of Interleukin-6-mediated immunometabolic reprogramming in COVID-19 and other viral infection-associated diseases. Int Immunopharmacol. 2022;110:109005. 35780641 10.1016/j.intimp.2022.109005 PMC9236983

[10] Sayah W Berkane I Guermache I. Interleukin-6, procalcitonin and neutrophil-to-lymphocyte ratio: potential immune-inflammatory parameters to identify severe and fatal forms of COVID-19. Cytokine. 2021;141:155428. 33550165 10.1016/j.cyto.2021.155428 PMC7834734

[11] Statsenko Y Al Zahmi F Habuza T. Impact of age and sex on COVID-19 severity assessed from radiologic and clinical findings. Front Cell Infect Microbiol. 2021;11:777070. 35282595 10.3389/fcimb.2021.777070 PMC8913498

[12] Alwani M Yassin A Al-Zoubi RM. Sex-based differences in severity and mortality in COVID-19. Rev Med Virol. 2021;31:e2223. 33646622 10.1002/rmv.2223 PMC8014761

[13] Pradhan A Olsson PE. Sex differences in severity and mortality from COVID-19: are males more vulnerable? Biol Sex Differ. 2020;11:53. 32948238 10.1186/s13293-020-00330-7 PMC7498997

[14] Broman N Rantasärkkä K Feuth T. IL-6 and other biomarkers as predictors of severity in COVID-19. Ann Med. 2021;53:410–2. 33305624 10.1080/07853890.2020.1840621 PMC7935117

[15] Han H Ma Q Li C. Profiling serum cytokines in COVID-19 patients reveals IL-6 and IL-10 are disease severity predictors. Emerg Microbes Infect. 2020;9:1123–30. 32475230 10.1080/22221751.2020.1770129 PMC7473317

[16] Akbari H Tabrizi R Lankarani KB. The role of cytokine profile and lymphocyte subsets in the severity of coronavirus disease 2019 (COVID-19): a systematic review and meta-analysis. Life Sci. 2020;258:118167. 32735885 10.1016/j.lfs.2020.118167 PMC7387997

[17] Motaib I Zbiri S Elamari S. Obesity and disease severity among patients with COVID-19. Cureus. 2021;13:e13165. 33717716 10.7759/cureus.13165 PMC7939537

[18] Gammone MA D’Orazio N. Review: obesity and COVID-19: a detrimental intersection. Front Endocrinol (Lausanne). 2021;12:652639. 33995281 10.3389/fendo.2021.652639 PMC8121172

[19] Hansen ESH Moeller AL Backer V. Severe outcomes of COVID-19 among patients with COPD and asthma. ERJ Open Res. 2021;7:00594–2020. 33527079 10.1183/23120541.00594-2020 PMC7701881

[20] Aveyard P Gao M Lindson N. Association between pre-existing respiratory disease and its treatment, and severe COVID-19: a population cohort study. Lancet Respir Med. 2021;9:909–23. 33812494 10.1016/S2213-2600(21)00095-3 PMC8016404

[21] Paliogiannis P Mangoni AA Dettori P. D-Dimer concentrations and COVID-19 severity: a systematic review and meta-analysis. Front Public Health. 2020;8:432. 32903841 10.3389/fpubh.2020.00432 PMC7438945

[22] Wool GD Miller JL. The impact of COVID-19 disease on platelets and coagulation. Pathobiology. 2021;88:15–27. 33049751 10.1159/000512007 PMC7649697

[23] Lin L Lu L Cao W. Hypothesis for potential pathogenesis of SARS-CoV-2 infection-a review of immune changes in patients with viral pneumonia. Emerg Microbes Infect. 2020;9:727–32. 32196410 10.1080/22221751.2020.1746199 PMC7170333

[24] Smilowitz NR Kunichoff D Garshick M. C-reactive protein and clinical outcomes in patients with COVID-19. Eur Heart J. 2021;42:2270–9. 33448289 10.1093/eurheartj/ehaa1103 PMC7928982

[25] Wannamethee SG Whincup PH Lennon L. Associations between fibrin D-dimer, markers of inflammation, incident self-reported mobility limitation, and all-cause mortality in older men. J Am Geriatr Soc. 2014;62:2357–62. 25516032 10.1111/jgs.13133 PMC4293158

[26] Kumar MP Mishra S Krishna Jha D. Coronavirus disease (COVID-19) and the liver: a comprehensive systematic review and meta-analysis. Hepatol Int. 2020;14:711–22. 32623633 10.1007/s12072-020-10071-9 PMC7335221

[27] Bajić D Matijašević J Andrijević L. Prognostic role of monocyte distribution width, CRP, procalcitonin and lactate as sepsis biomarkers in critically Ill COVID-19 patients. J Clin Med. 2023;12:1197. 36769843 10.3390/jcm12031197 PMC9917557

[28] Liu X Wang H Shi S. Association between IL-6 and severe disease and mortality in COVID-19 disease: a systematic review and meta-analysis. Postgrad Med J. 2022;98:871–9. 37063032 10.1136/postgradmedj-2021-139939

[29] Khalid A Ali Jaffar M Khan T. Hematological and biochemical parameters as diagnostic and prognostic markers in SARS-COV-2 infected patients of Pakistan: a retrospective comparative analysis. Hematology. 2021;26:529–42. 34334100 10.1080/16078454.2021.1950898

